# Predictors of low cardiac output syndrome after combined mitral and tricuspid valve surgery

**DOI:** 10.3389/fcvm.2024.1452820

**Published:** 2024-09-20

**Authors:** François Labaste, Yoan Lavie-Badie, Robin Bouchau, Lucie Boyère, Pascale Sanchez-Verlaan, Hélène Gonzalez, Bertrand Marcheix, Roxana Botea, Fanny Vardon-Bounes, Vincent Minville

**Affiliations:** ^1^ Department of Anesthesiology and Intensive Care, University Hospital of Toulouse, Toulouse, France; ^2^ RESTAURE, UMR 1301 Inserm—5070 CNRS—Université de Toulouse, Toulouse, France; ^3^ Department of Cardiology, Rangueil University Hospital, Toulouse, France; ^4^ Heart Valve Center, Toulouse University Hospital, Toulouse, France; ^5^ Department of Nuclear Medicine, Rangueil University Hospital, Toulouse, France; ^6^ Department of Cardiac Surgery, Rangueil University Hospital, Toulouse, France

**Keywords:** mitral regurgitation, mitral surgery, tricuspid surgery, low cardiac output syndrome (LCOS), cardiac surgery

## Abstract

**Introduction:**

Low cardiac output syndrome (LCOS) is a common complication in cardiac surgery, and we evaluated the incidence of its early occurrence after mitral and tricuspid combined cardiac surgery and its associated risk factors.

**Material and method:**

Retrospective, single institution study. We included 88 consecutive adult patients with severe mitral insufficiency scheduled for elective mitral and tricuspid valve replacement surgery between January 2015 and November 2018. The primary endpoint was the occurrence of LCOS, defined as the need for inotropic support or circulatory assistance after surgery. The secondary endpoint was mortality at 30 days.

**Results:**

LCOS occurred in 26 patients (29.5%) of the studied patients and its associated risk factors that appeared in the multivariate analysis were chronic kidney failure [adjusted odds ratio (OR) 3.1; 95% confidence interval (CI) 1.0–9.9, *p* = 0.05], pre-operative left ventricular heart failure (OR 5.3; 95% CI 1.3–10.9, *p* = 0.002), pre-operative right ventricular heart failure (OR 3.6; 95% CI 1.1–11.5, *p* = 0.02), and mitral valve replacement (OR 3.9; 95% CI 1.2–12.6, *p* = 0.03). LCOS affected the survival of patients (HR = 5.5; 95% CI 1.1–27.7 *p* = 0.04).

**Conclusion:**

LCOS is a frequent complication after mitral and tricuspid combined surgery and is associated with poor prognosis.

## 1 Introduction

Low cardiac output syndrome (LCOS) is a common complication in cardiac surgery and remains an important concern, associated with high morbidity and mortality (1, 2). It usually corresponds to a post-operative consideration of the cardiac function, which leads to the requirement of hemodynamic support by inotropic drugs or circulatory assistance (2).

Recent guidelines emphasized the need for combined tricuspid and mitral surgery when severe tricuspid regurgitation (TR) or mild to moderate TR with dilated tricuspid annulus (>40 mm or 21 mm^2^/m^2^) is associated with severe mitral disease (3). Previous evidence has shown that combined tricuspid and mitral surgery (CS) is associated with increased intrahospital mortality (4–6) but is necessary according to the worse evolution of a non-doing tricuspid surgery when it's needed (7–9). However, limited data are available to describe the perioperative risk, especially to describe the risk of post-operative LCOS (6, 10).

We made the hypothesis that LCOS is at the source of a large part of the perioperative mortality in patients who underwent CS and that risk factors could be identified. Therefore, the main objective of our study was to describe the incidence of early LCOS after mitral and tricuspid CS and to identify the factors associated with its occurrence.

## 2 Materials and methods

### 2.1 Ethics statement

A collection and computer processing of personal and medical data was implemented to analyze the results of the research. Toulouse University Hospital signed a commitment of compliance to the reference methodology MR-004 of the French National Commission for Informatics and Liberties (CNIL). After evaluation and validation by the data protection officer and according to the General Data Protection Regulation, this study on completing all the criteria was registered in the register of data study of the Toulouse University Hospital (RnIPH 2023-130) and covered by MR-004 (CNIL number: 2206723 v 0).

This study was approved by Toulouse University Hospital and it was confirmed that the ethical requirements were totally respected in the above report.

### 2.2 Study design and population

We retrospectively analyzed 88 consecutive adult patients with severe mitral insufficiency scheduled for elective mitral and tricuspid valve replacement surgery with cardiopulmonary bypass (CPB) in a single tertiary center between January 2015 and November 2018.

The inclusion criteria were adult patients (more than 18 years) with severe mitral insufficiency and with an indication for elective mitral valve surgery (mitral valve repair or replacement) combined with at least one surgical procedure on the tricuspid valve (tricuspid valve repair or replacement).

The exclusion criteria were patients with severe mitral insufficiency indicated for mitral valve surgery without a planned procedure on the tricuspid valve, patients with mitral stenosis, patients undergoing combined mitral and tricuspid surgery without severe mitral insufficiency in the pre-operative assessment, mitral and tricuspid surgery associated with other procedures (such as coronary artery bypass grafting or aortic valve surgery), and patients undergoing surgery for active endocarditis.

### 2.3 Objectives and outcome criteria

The primary endpoint was the occurrence of LCOS within the first 24 h after surgery, which was confirmed using at least one of the following:

• Implementation of temporary cardiac support such as extracorporeal life support (ECLS);
• Post-operative administration of a high dose of catecholamine with a positive inotropic effect: dobutamine (>5 μg/kg/min) and/or adrenaline (>0.1 μg/kg/min). The dose considered was the maximum dose administered for at least two consecutive hours.

The secondary endpoint was appreciated with all causes of mortality 30 days after heart surgery.

### 2.4 Perioperative care

All patients with mitral insufficiency scheduled for combined mitral and tricuspid surgery underwent a standardized pre-operative assessment. Specifically, they received transthoracic echocardiography and transesophageal echocardiography to confirm the severity of mitral insufficiency and to evaluate left and right ventricular function. In cases where associated aortic valve disease was detected, an additional aortic procedure could be considered, in accordance with guidelines. Coronary angiography was systematically performed, and coronary artery bypass grafting could be undertaken if a significant stenosis was discovered.

Mitral valve surgery could have been performed by mitral valve repair or mitral valve replacement (biological or mechanical). Similarly, tricuspid valve surgery could have been performed by valve repair or valve replacement. Valve repair (or plastic surgery) was chosen over replacement, according to recent recommendations (3). Valve replacement was performed only if repair was not feasible or insufficient at the end of the surgery. In all cases, the choice of surgical technique was made considering ultrasound and anatomical data, age, predisposition, and patient preferences.

Surgery was performed under general anesthesia with induction by propofol, sufentanil, and cisatracurium and maintenance by sevoflurane before the start of CPB and by propofol with an electric syringe pump during CPB. The depth of sedation was monitored by a bispectral index. Labile blood products were transfused according to the department protocol. During CPB, a transfusion threshold of 8 g/dl was set, and then, outside of CPB, a threshold of 8.5 g/dl was set. During CPB, patients received antegrade cardioplegia with cold oxygenated blood at 4°C, along with potassium chloride and magnesium sulfate administration. Cardioplegia was then maintained with retrograde cold oxygenated blood cardioplegia.

At the end of surgery, patients were transferred to cardiovascular intensive care for follow-up management. Overall management was in line with standards of clinical practice.

From the weaning of the CPB, the administration of inotropic drugs was not protocolized and left to the discretion of the physicians. However, even in the absence of protocols, the initiation of inotropic therapy was done in the presence of a cluster of clinical and biological arguments for low cardiac output, confirmed by echocardiography.

### 2.5 Data collection

All data were obtained from the clinical and biological information management systems at the Toulouse University Hospital.

Patients with a pre-operative glomerular filtration rate [estimated by the Modification of Diet in Renal Disease (MDRD) formula] of less than 60 ml/min/1.73 m^2^ were considered to have chronic renal failure. The notion of ischemic heart disease implied a history of angioplasty/coronary stent or coronary bypass surgery.

The pre-operative echocardiographic data provided information concerning right and left ventricular functions.

Left ventricular failure was defined as left ventricular ejection fraction (LVEF) less than 50% (11).

Right ventricular function was categorized as follows (11, 12):

- normal right heart function,
- right ventricular failure [tricuspid annular plane systolic excursion (TAPSE) < 15 mm and/or S wave < 9.5 cm/s and/or severe PAH with measured PAPS > 70 mmHg], and
- non-evaluated right ventricular function.

Post-operative acute renal failure was defined according to the Kidney Disease Improving global outcomes (KDIGO) criteria (13). Serum creatinine concentration values were recorded before and daily after surgery. Acute Kidney Injury (AKI) stage 1 was defined as a rise in serum creatinine levels ≥26.4 µmol/L within 48 h or an increase to 1.5–1.9 times of the baseline value within 7 days. AKI stage 2 was defined as an increase in serum creatinine levels to 2.0–2.9 times of the baseline value, while AKI stage 3 was defined as an increase in serum creatinine levels to ≥3 times of the baseline value, an absolute increase in serum creatinine levels of ≥354 µmol/L, or the initiation of renal replacement therapy (RRT).

Mitral regurgitations (MRs) were classified into three categories: primary, functional, and ischemic mitral insufficiency (3). Primary lesions were defined by the presence of a structural abnormality of the mitral valve, such as prolapse, flail, or thickening of the leaflets. Functional or secondary mitral regurgitation were defined by the presence of left ventricular remodeling or dilation, leading to mitral valve dysfunction without primary structural abnormalities of the valve itself. Ischemic mitral lesions were defined by the presence of mitral regurgitation due to ischemic heart disease, typically associated with left ventricular dysfunction or papillary muscle displacement following a myocardial infarction. The type of mitral regurgitation was determined pre-operatively by the cardiologist based on the results of the various echocardiograms.

### 2.6 Statistical method

In the first step of descriptive analysis, the population was characterized. The continuous variables were expressed as median and interquartile range. The qualitative variables were expressed in absolute numbers and percentages.

A univariate analysis was performed for the main outcome criterion and then for the secondary outcome criterion after separating the study population according to whether or not the endpoint was reached. Within this analysis, the continuous variables were compared using a non-parametric Mann–Whitney *U* test. The qualitative variables were compared using the χ^2^ test or Fisher's exact test. In addition, we attempted to define thresholds for the quantitative variables. The relevant threshold value was defined using an ROC curve to analyze the sensitivity and specificity of the different values.

We evaluated the association between the different covariates and the variable that was explained (composite criterion of post-operative LCOS) in the multivariate analysis (logistic regression) by measuring the odds ratio (OR). A backward elimination procedure was used by including all variables with a *p* < 0.1 and then gradually eliminating non-significant variables. A *p* < 0.05 was considered statistically significant. The analyses were carried out on XLSTAT® software version 2018 (Addison 2018, XLSTAT Statistical and Data Analysis Solution, Paris, France).

## 3 Results

### 3.1 General characteristics of the population

From January 2015 to November 2018, 88 patients underwent combined mitral and tricuspid valve surgery and were included in our study. All included patients had secondary tricuspid insufficiency. Their main characteristics are summarized in Table 1.

**Table 1:** Baseline characteristics of the population.

General characteristics	Total *N* = 88	Without LOCS *N* = 62	With LOCS *N* = 26	*p*
Age (years)	69 (63–75)	69 (62–75)	70 (65–77)	0.405
Male sex, % (*n*)	72.7 (64)	72.6 (45)	73.1 (19)	0.961
Weight (kg)	73 (65–86)	70 (64–89)	77 (68–80)	0.848
Size (m)	1.70 (1.64–1.77)	1.70 (1.65–1.77)	1.70 (1.63–1.75)	0.484
Previous cardiac surgery, % (*n*)	9.1 (8)	6.5 (4)	15.4 (4)	0.413
Hypertension, % (*n*)	48.9 (43)	50.0 (31)	42.6 (12)	0.742
Peripheral vascular disease, % (*n*)	3.4 (3)	3.2 (2)	3.8 (1)	0.884
Atrial fibrillation, % (*n*)	64.8 (57)	62.9 (39)	69.2 (18)	0.571
Diabetes mellitus, % (*n*)	15.9 (14)	12.9 (8)	23.1 (6)	0.234
Coronary disease with previous PCI, % (*n*)	9,1 (8)	3.2 (2)	23.1 (6)	**0**.**003**
Previous endocarditis, % (*n*)	2.3 (2)	1.6 (1)	3.8 (1)	0.522
Chronic pulmonary disease, % (*n*)	22.7 (20)	21.0 (13)	26.9 (7)	0.543
Chronic renal failure, % (*n*)	44.3 (39)	33.9 (21)	69.2 (18)	**0**.**002**
Stroke, % (*n*)	9.1 (8)	9.7 (6)	7.7 (2)	0.766
Pre-operative evaluation				
Hemoglobin (g/dl)	13.1 (12.1–14.1)	13.0 (12.2–14.1)	13,3 (11.9–14.3)	0.691
NYHA class III or IV, % (*n*)	34.1 (30)	27.4 (17)	50.0 (13)	0.164
LVEF (%)	58 (50–65)	60 (55–65)	51 (45–60)	**0**.**014**
LVEF < 50% (LV failure), % (*n*)	18.2 (16)	9.7 (6)	38.5 (10)	**<0.001**
RV function, % (*n*)				**0**.**023**
Normal	56.8 (50)	62.9 (39)	42.3 (11)	
RV failure	29.5 (26)	21.0 (13)	50.0 (13)	
Not assessed	12.4 (12)	15,1 (10)	6.3 (2)	
Functional MR, % (*n*)	50.0 (44)	50.0 (31)	50.0 (13)	0.999
Primitive MR, % (*n*)	43.2 (38)	45.2 (28)	38.5 (10)	0.563
Ischemic MR, % (*n*)	6.8 (6)	4.8 (3)	11.5 (3)	0.255
EuroSCORE II (%)	2.7 (1.8–4.2)	2.5 (1.7–3.7)	3.6 (2.3–5.8)	**0**.**024**
Perioperative data				
Aortic clamping time (min)	64 (54–82)	62 (51–79)	73 (55–88)	0.344
CPB assistance time (min)	19 (13–25)	17 (12–22)	24 (15–28)	**0**.**027**
CPB duration (min)	93 (77–116)	89 (75–114)	106 (83–120)	0.125
Mitral replacement, % (*n*)	47.7 (42)	40.3 (25)	65.3 (17)	**0**.**032**
Red blood transfusion, % (*n*)	13.6 (12)	9.7 (6)	23.1 (6)	0.095

LVEF, left ventricular ejection fraction; MR, mitral regurgitation; NYHA, New York Heart Association; LV, left ventricular; RV, right ventricular; CPB, cardiopulmonary bypass; PCI, percutaneous coronary intervention.

Data are expressed as percentage (*n*) or median (interquartile range). Differences were considered statistically significant for *p* < 0.05 (in bold).

Most patients were male (*n* = 64, 72.7%) with a median age of 69 (IQR 63–75) years. The median EuroSCORE II was 2.7% (IQR 1.8–4.2). Two patients (2.3%) had already undergone cardiac surgery.

All the patients who were included had a pre-operative assessment of left heart function with echocardiography. The median LVEF was 58% (IQR 50–65), and 16 patients (18.2%) had left ventricular failure. A total of 12 patients (12.4%) did not have evaluation of the right cardiac function in the pre-operative period, and 26 patients (29.5%) had right ventricular failure.

Furthermore, 42 patients (47.7%) received a mitral valve replacement, and 46 patients (52.3%) had a mitral valve repair. A concomitant tricuspid valve repair was performed in 100% of cases, and no patient underwent tricuspid valve replacement.

### 3.2 Low cardiac output syndrome

A total of 26 (29.5%) patients experienced LCOS. Of these, 5 patients received cardiac support with ECLS, 10 patients received more than 0.1 µg/kg/min of adrenaline, 6 patients received more than 5 μg/kg/min of dobutamine, and 5 patients received both adrenaline and dobutamine during the first 24 h after surgery.

On admission to the intensive care unit following surgery (D0), it was observed that lactates were significantly higher in the group with LCOS (4.6 vs. 1.2 mmol/L at D0, *p* < 0.001).

After univariate analysis, the best logistic regression model identified four independent risk factors for LCOS: chronic renal failure (OR = 3.1; CI 1.0–9.9, *p* = 0.05), pre-operative left ventricular failure (OR = 5.3; CI 1.3–20.9, *p* = 0.002), pre-operative right ventricular failure (OR = 3.5; CI 1.1–11.5, *p* = 0.02), and mitral valve replacement vs. mitral valve repair (OR = 3.9; CI 1.2–12.6 *p* = 0.03) (Table 2).

**Table 2:** Risk factors of LCOS.

	Odds ratio	95% CI	*p*
Chronic renal failure	3.14	1.0–9.9	**0**.**05**
Pre-operative LV failure	5.26	1.3–20.9	**0**.**002**
Pre-operative RV failure	3.57	1.1–11.5	**0**.**02**
Mitral replacement	3.87	1.2–12.6	**0**.**03**
Hosmer–Lemeshow test	0.99		
% of patients well classified by model	77.3%		
AUC of the model	0.82		

CI, confidence interval; LV, left ventricular; RV, right ventricular; AUC, area under the curve.

Differences were considered statistically significant for *p* < 0.05 (in bold).

### 3.3 LCOS: post-operative outcomes and mortality at 30 days

The occurrence of LCOS was associated with an increased incidence of post-operative complications. Data on post-operative outcome in the ICU are summarized in Table 3. During the first 30 days after surgery, 9 (10.2%) patients died. The median length of stay in intensive care was 5 (IQR 3–7) days. The incidence of acute renal failure is increased in cases of LOCS [22.6% (14 patients) vs. 61.5% (16 patients) group LOCS, *p* = 0.007]. All KDIGO classes are affected.

**Table 3:** Post-operative outcomes.

	Total *N* = 88	Without LCOS *N* = 62	With LCOS *N* = 26	*p*
SAPS2	30 (24–39)	28 (23–37)	35 (29–52)	**0**.**013**
Acute renal failure, % (*n*)	34.1 (30)	22.6 (14)	61.5 (16)	**0**.**007**
KDIGO 1	17.0 (15)	12.9 (8)	26.9 (7)	
KDIGO 2	8.0 (7)	6.5 (4)	11.5 (3)	
KDIGO 3	9.1 (8)	3.2 (2)	23.1 (6)	
Renal replacement therapy, % (*n*)	9.1 (8)	3.2 (2)	23.1 (6)	**0**.**003**
Troponin level pic (ng/L)	1,460 (928–2,456)	1,165 (766–225)	2,012 (1,502–3,193)	**0**.**001**
Post-operative atrial fibrillation, % (*n*)	58.0 (51)	54.8 (34)	65.4 (17)	0.361
Lactatemia D0 (mmol/L)	1.7 (1.2–3.7)	1.2 (1.1–1.7)	4.6 (2.0–9.0)	**<0.001**
Lactatemia D1 (mmol/L)	4.2 (2.8–5.6)	3.3 (2.0–4.8)	4.9 (2.9–7.5)	0.101
SvO2 D0 (%)	80 (72–83)	76 (71–80)	83 (81–85)	**0**.**011**
SvO2 D1 (%)	70 (66–75)	70 (62–71)	70 (67–75)	0.188
Mechanical ventilation (h)	9 (5–17)	7 (5–11)	44 (11–125)	**<0.001**
Re-intubation, % (*n*)	10.2 (9)	3.2 (2)	26.9 (7)	**<0.001**
Pneumoniae, % (*n*)	27.3 (24)	19.4 (12)	46.2 (12)	**0**.**010**
Reintervention, % (*n*)	21.5 (18)	15.1 (11)	30.8 (8)	0.175
Post-operative red blood transfusion, % (*n*)	39.8 (35)	32.3 (20)	57.7 (15)	**0**.**026**
Norepinephrin infusion duration (days)	2 (1–4)	2 (1–3)	4 (3–5)	**<0.001**
Epinephrin infusion duration (days)	0 (0–1)	0 (0–0)	3 (0–4)	**<0.001**
Dobutamin infusion duration (days)	0 (0–2)	0 (0–2)	2 (0–3)	**0**.**007**
ICU length of stay (days)	5 (3–7)	5 (2–7)	7 (5–9)	**0**.**010**
Hospitalization length of stay (days)	15 (11–21)	15 (11–20)	17 (12–29)	0.204
Mortality (day 30), % (*n*)	10.2 (9)	3.2 (2)	26.9 (7)	**<0.001**

LCOS, low cardiac output syndrome; SAPS2, Simplified Acute Physiology Score 2; ICU, intensive care unit.

Data are expressed as percentage (*n*) or median (interquartile range). Differences were considered statistically significant for *p* < 0.05 (in bold).

**Figure 1:**
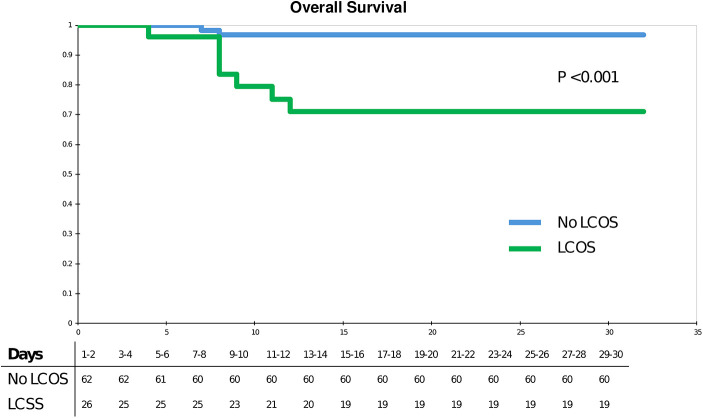
Kaplan–Meier analysis.

Nine patients died during the post-operative 30 days. Post-operative LCOS, as defined for the primary endpoint, affected the survival of patients who had combined mitral and tricuspid valve surgery (Figure 1): 2 (3.2%) patients died in the group without LCOS, vs. 7 (26.9%) in the group with (*p* < 0.001). Patient characteristics and univariate analysis were detailed in Supplementary Table S1.

Risk factors for mortality within 30 days post-operatively were: LCOS (HR = 5.5; 95% CI 1.1–27.7, *p* = 0.04) and Simplified Acute Physiology Score 2 (SAPS2) > 44 (HR = 8.7; CI 1.7–44.2; *p* = 0.009) (Table 4).

**Table 4:** Cox regression of post-operative mortality at day 30.

	Hazard ratio	95% CI	*p*
LCOS	5.5	1.1–27.7	**0**.**04**
SAPS2 > 44	8.7	1.7–44.2	**0**.**009**

CI, confidence interval; LCOS, low cardiac output syndrome; SAPS2, Simplified Acute Physiology Score 2.

Differences were considered statistically significant for *p* < 0.05 (in bold).

## 4 Discussion

The main results of this study can be summarized as follows: LCOS after combined mitral and tricuspid valve surgery is frequent and has an impact on mortality. Chronic renal failure, reduced left ventricular function less than 50%, the use of a mitral prosthesis over repair, and pre-operative right ventricular failure were the main factors associated with its occurrence. LCOS was an independent risk factor for early mortality.

Despite a significant decline in cardiac surgery-associated mortality, post-operative LCOS remains a frequent source of concern (14). Indeed, a study on a large consecutive series of 3,039 patients undergoing mitral valve replacement or repair over an 18-year period found that the prevalence of LCOS has decreased over time but that its overall prevalence remained at 7% (6). It was slightly lower in the case of isolated aortic replacement in a study on a series of 2,255 patients that reported a prevalence of 3.9% (1). Over the past decade, there has been a considerable increase in the volume of combined mitral and tricuspid valve surgeries. Indeed, the recent guidelines (3, 15) suggest that, when performing a mitral surgery, the need for associated tricuspid repair should be systematically considered. The tricuspid ring size (>40 mm or 21 mm/m^2^) or significant regurgitation are the main indications. There is scarce data on the prevalence of LCOS in this situation. We found a prevalence of 29.5% in our cohort, which is far higher than what is described for isolated mitral surgery. Early mortality was 10.2%, a rate comparable to those observed in recent literature (5). LCOS was associated with prognosis, which is consistent with the available evidence (2). While these data are worthy of confirmation by other studies, this is undoubtedly a high-risk situation, for which the identification of at-risk subjects is of paramount importance.

We found four independent factors that influence the occurrence of LCOS. Its most common pathophysiological approach is ischemic/reperfusion injury of the heart. By consequence, the contributing factors include pre-operative myocardial dysfunction, degree of myocardial protection, systemic inflammatory responses, and alterations in signal transduction systems. Coherently, the pre-operative left ventricular function is a well-known predictor of LCOS (1, 2, 14). Renal failure is widely reported as a major risk factor in cardiac valve operations (16). Interestingly, the use of a mitral prosthesis over repair was a predictor of LCOS. This has already been described elsewhere (14) and emphasizes the importance of the architecture of the mitral valve apparatus (both leaflets, chordae, and papillary muscle) in the left ventricular mechanics (17). Finally, the most challenging and critical point in right heart valve surgery is the pre-operative right ventricular function. As the right ventricular function is a complex interplay between preload, afterload, and contractility, we merged contractility parameters as TAPSE or S wave with pulmonary hypertension. However, it is particularly interesting to note that these data were not available for all patients, unlike LVEF, proof of the paucity of interest in the right ventricle function in the pre-operative study (9). Our results highlight the need for a comprehensive echocardiographic assessment of right ventricular parameters prior to the combined surgery to identify patients at risk (18).

Cardiac support time was significantly longer in univariate analysis in the group of patients with post-operative LCOS. This time corresponds with the time required for weaning from CPB. We considered that this period was not a risk factor but a consequence of the dysfunction. In fact, in the event of failure to recover adequate cardiac function, CPB weaning is slowed down and the time of cardiac assistance extended (19).

Consequently, our results allowed us to identify patients at risk. Considering that mitral valve disease is chronic in most of the cases and could benefit from scheduled surgery, these data can guide us in both the selection and pre-operative optimization of the status of the patients concerned. In fact, without knowing the exact level of risk, it can be assumed that the accumulation of non-modifiable risk factors in the same patient provides arguments in favor of a poor post-operative hemodynamic progression and a decrease in short-term survival. New scores are now available to support the decision-making process (20).

In addition, the importance of medical treatment of chronic heart failure to obtain the best possible right and left heart function at the time of surgery should be emphasized. Levosimendan could be considered in these patients. Pre-operative administration of levosimendan is not associated with improved post-operative outcomes after cardiac surgery; however, the proportion of patients included for double mitral and tricuspid repair was small (21, 22). Thus, the efficacy of pre-operative administration of levosimendan in reducing the risk of LCOS in this particular category of patients remains unclear. An ongoing randomized trial will help answer this question (clinicaltrial.gov; NCT05233202).

Our work has several limitations.

First, this is a retrospective study. Some of the data that could be useful in predicting the occurrence of our main outcome criterion were missing. Some of the pre-operative ultrasound data were not available. Although all LVEFs were found, it appears that some of the data required to assess right heart function were not available. However, we decided to integrate this data into our analysis. We did not find an association between the risk of post-operative LCOS and the lack of pre-operative information on right heart function.

Some data collected had a significant *p* value only in the univariate analysis. This may be caused by a lack of power in our study. A larger study may find stronger results and other risk factors associated with LCOS occurrence.

## 5 Conclusion

Post-operative low cardiac output syndrome after mitral and tricuspid combined surgery is frequent and associated with a poor prognosis. Pre-operative screening of its predictive factors, right ventricular function in particular, is mandatory.

## Data Availability

The original contributions presented in the study are included in the article/**Supplementary Material**, further inquiries can be directed to the corresponding author.

## References

[1] Maganti MD Rao V Borger MA Ivanov J David TE. Predictors of low cardiac output syndrome after isolated aortic valve surgery. Circulation. (2005) 112:I448–452. 10.1161/CIRCULATIONAHA.104.526087 16159861

[2] Lomivorotov VV Efremov SM Kirov MY Fominskiy EV Karaskov AM. Low-cardiac-output syndrome after cardiac surgery. J Cardiothorac Vasc Anesth. (2017) 31:291–308. 10.1053/j.jvca.2016.05.029 27671216

[3] Vahanian A Beyersdorf F Praz F Milojevic M Baldus S Bauersachs J 2021 ESC/EACTS guidelines for the management of valvular heart disease. Eur Heart J. (2022) 43:561–632. 10.1093/eurheartj/ehab395 34453165

[4] Alsoufi B Rao V Borger MA Maganti M Armstrong S Feindel CM Scully HE David TE. Short- and long-term results of triple valve surgery in the modern era. Ann Thorac Surg. (2006) 81:2172–7; discussion 2177–8. 10.1016/j.athoracsur.2006.01.072 16731149

[5] Ohmes LB Kim L Feldman DN Lau C Munjal M Di Franco A Contemporary prevalence, in-hospital outcomes, and prognostic determinants of triple valve surgery: national database review involving 5,234 patients. Int J Surg. (2017) 44:132–8. 10.1016/j.ijsu.2017.06.046 28642087

[6] Badhwar V Rankin JS He M Jacobs JP Furnary AP Fazzalari FL Performing concomitant tricuspid valve repair at the time of mitral valve operations is not associated with increased operative mortality. Ann Thorac Surg. (2017) 103:587–93. 10.1016/j.athoracsur.2016.06.004 27570159

[7] Ruel M Rubens FD Masters RG Pipe AL Bédard P Mesana TG. Late incidence and predictors of persistent or recurrent heart failure in patients with mitral prosthetic valves. J Thorac Cardiovasc Surg. (2004) 128:278–83. 10.1016/j.jtcvs.2003.11.048 15282466

[8] Nath J Foster E Heidenreich PA. Impact of tricuspid regurgitation on long-term survival. J Am Coll Cardiol. (2004) 43:405–9. 10.1016/j.jacc.2003.09.036 15013122

[9] Chikwe J Itagaki S Anyanwu A Adams DH. Impact of concomitant tricuspid annuloplasty on tricuspid regurgitation, right ventricular function, and pulmonary artery hypertension after repair of mitral valve prolapse. J Am Coll Cardiol. (2015) 65:1931–8. 10.1016/j.jacc.2015.01.059 25936265

[10] Leone A Fortuna D Gabbieri D Nicolini F Contini GA Pigini F Triple valve surgery: results from a multicenter experience. J Cardiovasc Med (Hagerstown). (2018) 19:382–8. 10.2459/JCM.0000000000000665 29877976

[11] McDonagh TA Metra M Adamo M Gardner RS Baumbach A Böhm M 2021 ESC guidelines for the diagnosis and treatment of acute and chronic heart failure. Eur Heart J. (2021) 42:3599–726. 10.1093/eurheartj/ehab368 34447992

[12] Sun X Zhang H Aike B Yang S Yang Z Dong L Tricuspid annular plane systolic excursion (TAPSE) can predict the outcome of isolated tricuspid valve surgery in patients with previous cardiac surgery? J Thorac Dis. (2016) 8:369–74. 10.21037/jtd.2016.02.38 27076931 PMC4805791

[13] Khwaja A. KDIGO clinical practice guidelines for acute kidney injury. Nephron Clin Pract. (2012) 120:c179–84. 10.1159/000339789 22890468

[14] Maganti M Badiwala M Sheikh A Scully H Feindel C David TE Predictors of low cardiac output syndrome after isolated mitral valve surgery. J Thorac Cardiovasc Surg. (2010) 140:790–6. 10.1016/j.jtcvs.2009.11.022 20152992

[15] Baumgartner H Falk V Bax JJ De Bonis M Hamm C Holm PJ 2017 ESC/EACTS guidelines for the management of valvular heart disease. Eur Heart J. (2017) 38:2739–91. 10.1093/eurheartj/ehx391 28886619

[16] Jamieson WRE Edwards FH Schwartz M Bero JW Clark RE Grover FL. Risk stratification for cardiac valve replacement. National Cardiac Surgery Database. Ann Thorac Surg. (1999) 67:943–51. 10.1016/S0003-4975(99)00175-7 10320233

[17] Coutinho GF Antunes MJ. Mitral valve repair for degenerative mitral valve disease: surgical approach, patient selection and long-term outcomes. Heart. (2017) 103:1663–9. 10.1136/heartjnl-2016-311031 28566474

[18] Mattei A Strumia A Benedetto M Nenna A Schiavoni L Barbato R Perioperative right ventricular dysfunction and abnormalities of the tricuspid valve apparatus in patients undergoing cardiac surgery. J Clin Med. (2023) 12:7152. 10.3390/jcm12227152 38002763 PMC10672350

[19] Milojevic M Milosevic G Nikolic A Petrovic M Petrovic I Bojic M Mastering the best practices: a comprehensive look at the European guidelines for cardiopulmonary bypass in adult cardiac surgery. J Cardiovasc Dev Dis. (2023) 10:296. 10.3390/jcdd10070296 37504552 PMC10380276

[20] Dreyfus J Bohbot Y Coisne A Lavie-Badie Y Flagiello M Bazire B Predictive value of the TRI-SCORE for in-hospital mortality after redo isolated tricuspid valve surgery. Heart. (2023) 109:951–8. 10.1136/heartjnl-2022-322167 36828623

[21] Guarracino F Heringlake M Cholley B Bettex D Bouchez S Lomivorotov VV Use of levosimendan in cardiac surgery: an update after the LEVO-CTS, CHEETAH, and LICORN trials in the light of clinical practice. J Cardiovasc Pharmacol. (2018) 71:1–9. 10.1097/FJC.0000000000000551 29076887 PMC5768218

[22] Cholley B Bojan M Guillon B Besnier E Mattei M Levy B Overview of the current use of levosimendan in France: a prospective observational cohort study. Ann Intensive Care. (2023) 13:69. 10.1186/s13613-023-01164-3 37552372 PMC10409690

