# Supplementary material for: Predictors of low cardiac output syndrome after combined mitral and tricuspid valve surgery
Source: Front Cardiovasc Med. 2024 Sep 20;11:1452820. doi: 10.3389/fcvm.2024.1452820 (PMC11449694; doi:10.3389/fcvm.2024.1452820)
Supplement: Supplementary file 1 [file Table1.docx]

Supplementary:

Table S1: Risk factor of mortality at D30, univariate analysis

|  | **Alive at D30**  **N = 79** | **Dead at D30**  **N = 9** | ***p*** |
| --- | --- | --- | --- |
| **General characacteristics** |  |  |  |
| Age (years) | 68 [63-75] | 74 [71-81] | 0.405 |
| Male sex, %(n) | 73.4 (58) | 66.7 (6) | 0.667 |
| Weight (kg) | 74 [65-87] | 70 [68-80] | 0.848 |
| Size (m) | 1.70 [1.64-1.77] | 1.68 [1.66-1.75] | 0.484 |
| Previous cardiac surgery, %(n) | 8.9 (7) | 11.1 (1) | 0.778 |
| Hypertension, %(n) | 50.6 (40) | 33.3 (3) | 0.325 |
| Peripheral vascular disease, %(n) | 3.8 (3) | 0.0 (0) | 0.552 |
| Atrial fibrillation, %(n) | 65.8 (52) | 55.6 (5) | 0.541 |
| Diabetes mellitus, %(n) | 16.5 (13) | 11.1 (1) | 0.678 |
| Coronary disease with previous PCI, %(n) | 7.6 (6) | 22.2 (2) | 0.148 |
| Previous endocarditis, %(n) | 2.3 (2) | 0.0 (0) | 0.629 |
| Chronic pulmonary disease, %(n) | 24.1 (19) | 11.1 (1) | 0.380 |
| Chronic renal failure, %(n) | 41.8 (33) | 66.7 (6) | 0.153 |
| Stroke, %(n) | 8.9 (7) | 11.1 (1) | 0.824 |
| Haemoglobin, g/dl | 13.1 [12.2-14.2] | 13.0 [11.6-13.6] | 0.691 |
| NYHA III ou IV, %(n) | 38.1 (30) | 44.4 (4) | 0.795 |
| LVEF, % | 57 [50-65] | 60 [50-70] | **0.014** |
| LV failure (LVEF < 50%), %(n) | 19.0 (15) | 11.1 (1) | 0.561 |
| RV function |  |  | 0.346 |
| Normal | 57.0 (45) | 55.6 (5) |  |
| RV failure | 27.8 (22) | 44.4 (4) |  |
| Not assessed | 15.2 (12) | 0.0 (0) |  |
| Functional MR, %(n) | 49.4 (39) | 55.6 (5) | 0.725 |
| Primitive MR , %(n) | 43.0 (38) | 44.4 (4) | 0.936 |
| Ischemic MR, %(n) | 7.6 (6) | 0.0 (0) | 0.392 |
| EuroSCORE II (%) | 2.6 [1.8-4.2] | 3.3 [2.9-4.0] | **0.024** |
| **Perioperative data** |  |  |  |
| Aortic clamping time (min) | 63[53-82] | 72 [54-82] | 0.343 |
| CPB assistance time (min) | 17 [13-24] | 25 [21-27] | **0.027** |
| CPB duration (min) | 91 [77-117] | 99 [95-112] | 0.125 |
| Mitral replacement, %(n) | 45.6 (36) | 66.7 (6) | 0.230 |
| Red blood transfusion, %(n) | 12.7 (10) | 22.2 (2) | 0.428 |
| **Postoperative outcomes** |  |  |  |
| Lactatemia J0 (mmol/l) | 1.7 [1.2-3.1] | 2.0 [1.4-4.3] | **< 0.0001** |
| Lactatemia J1 (mmol/l) | 3.3 [2.6-5.3] | 5.8 [4.7-9.2] | 0.101 |
| ScvO2 J0 (%) | 79.2 [72.1-83.1] | 85.1 [83.0-85.7] | **0.011** |
| ScvO2 J1 (%) | 70.0 [65.7-73.6] | 72.6 [66.0-75.3] | 0.188 |
| SAPS2 | 29 [24-37] | 52 [45-68] | **0.013** |
| Acute renal failure, %(n) |  |  | **0.0002** |
| KDIGO 1 | 19.0 (15) | 0.0 (0) |  |
| KDIGO 2 | 6.3 (5) | 11.1 (1) |  |
| KDIGO 3 | 3.8 (3) | 66.7 (6) |  |
| Renal Replacement Therapy, %(n) | 3.8 (3) | 66.7 (6) | **< 0.0001** |
| LCOS | 24.1 (19) | 77.8 (7) | **< 0.001** |
| Troponin level pic (ng/l) | 1374 [800-2409] | 2066 [1756-3324] | **0.001** |
| Mechanical ventilation duration, (hours) | 7 [5-13] | 111 [59-173] | **< 0.0001** |
| Re-intubation | 6.3 (5) | 44.4 (4) | **0.0003** |
| Pneumoniae | 24.1 (19) | 55.6 (5) | **0.044** |
| Reintervention | 20.3 (16) | 33.3 (3) | 0.366 |
| Postoperative Red blood transfusion, %(n) | 39.7 (35) | 66.7 (6) | 0.081 |

Data are expressed as percentages (n) or median (interquartile); LVEF: left ventricular ejection fraction; MR: mitral regurgitation; NYHA: New York Heart Association; LV: Left Ventricular; RV : Right Ventricular, CPB :Cardiopulmonary bypass, SvO2: central mixed venous oxygen saturation; SAPS 2 : Simplified Acute Physiology Score2; LCOS: Low cardiac output syndrome; ECLS: Extracorporeal Life Support; PCI : Percutaneous coronary intervention

Differences were considered statistically significant for p < 0,05.
